# The dynamics of human behavior in the public goods game with institutional incentives

**DOI:** 10.1038/srep28809

**Published:** 2016-06-24

**Authors:** Yali Dong, Boyu Zhang, Yi Tao

**Affiliations:** 1 School of Statistics and Institute of National Accounts, Beijing Normal University, Beijing, China; 2 Laboratory of Mathematics and Complex Systems, Ministry of Education, School of Mathematical Sciences, Beijing Normal University, Beijing, China; 3 Key Lab of Animal Ecology, Institute of Zoology, Chinese Academy of Sciences, Beijing, China

## Abstract

The empirical research on the public goods game (PGG) indicates that both institutional rewards and institutional punishment can curb free-riding and that the punishment effect is stronger than the reward effect. Self-regarding models that are based on Nash equilibrium (NE) strategies or evolutionary game dynamics correctly predict which incentives are best at promoting cooperation, but individuals do not play these rational strategies overall. The goal of our study is to investigate the dynamics of human decision making in the repeated PGG with institutional incentives. We consider that an individual’s contribution is affected by four factors, which are self-interest, the behavior of others, the reaction to rewards, and the reaction to punishment. We find that people on average do not react to rewards and punishment, and that self-interest and the behavior of others sufficiently explain the dynamics of human behavior. Further analysis suggests that institutional incentives promote cooperation by affecting the self-regarding preference and that the other-regarding preference seems to be independent of incentive schemes. Because individuals do not change their behavioral patterns even if they were not rewarded or punished, the mere potential to punish defectors and reward cooperators can lead to considerable increases in the level of cooperation.

In the public goods game (PGG), the only Nash equilibrium (NE) that is based on monetary considerations is for all players to free ride. However, in PGG experiments, most individuals contributed approximately half of their endowment to the public pool, and this contribution tends to decrease as individuals play the game repeatedly^1^,^2^,^3^,^4^. Much research has been devoted to explain the dynamics of human behavior in PGG. Early investigations assumed that individuals consider self-interests only and interpreted the decline in cooperation as a preference for free-riding, which pursues higher payoffs or frustrates attempts at kindness^5^,^6^,^7^. However, even in one-shot PGG experiments, many people preferred to cooperate provided that others also cooperate, which is inconsistent with the self-interest argument^8^,^9^,^10^,^11^. Subsequent studies indicated that such conditionally cooperative behavior can be explained by other-regarding preferences (e.g., inequity aversion or conformity), where a conditional cooperator (or conformist) changes his/her contribution in the next round in the direction of the average group contribution of the current round^8^,^9^,^10^,^11^,^12^,^13^,^14^,^15^,^16^. In repeated PGG, conditional cooperators who observe others free-riding will reduce their contributions, which leads to a decline in cooperation. Recently, Fischbacher and Gächter^17^ indicated that human behaviors in PGG can be better described through a combination of the self-regarding preference and the other-regarding preference. They showed that most people are imperfect conditional cooperators who match others’ contributions only partly. This behavior implies that the voluntary cooperation in PGG is inherently fragile. Even if there are no free riders in the group, imperfect conditional cooperators decrease their contribution because of the self-regarding preference. Fischbacher and Gächter^17^ then suggested that other mechanisms such as punishment and rewards are necessary to sustain cooperation.

Much of the empirical and theoretical research on PGG indicates that rewards and/or punishment can curb free-riding. One class of literature has addressed the so-called peer (or decentralized) incentives, where players can impose fines or bonuses on others at a cost to themselves^2^,^18^,^19^,^20^,^21^,^22^,^23^,^24^,^25^,^26^,^27^,^28^,^29^. Another class of literature considered the so-called institutional (or centralized) incentives. In this scenario, it is not individuals who reward or punish but rather an institution that rewards and punishes individuals based on their contributions^13^,^15^,^30^,^31^,^32^,^33^,^34^,^35^,^36^,^37^,^38^,^39^,^40^,^41^,^42^,^43^,^44^. For both peer and institutional incentives, theoretical studies have determined that the effect of rewards is not equivalent to the effect of punishment^7^,^18^,^24^,^25^,^26^,^27^,^28^,^29^,^31^,^34^,^35^,^36^,^37^,^38^,^40^,^41^,^42^,^43^,^44^. Specifically, punishment can eliminate selfish behaviors in a cooperative population and stabilize cooperation^18^,^24^,^25^,^27^,^31^,^34^,^35^,^36^,^37^,^38^,^41^,^42^. By contrast, rewards play an important role in leaving the selfish state but are relatively ineffective in maintaining a high cooperation level^18^,^25^,^26^,^28^,^29^,^35^,^37^,^38^,^42^. The asymmetry between reward and punishment has also been observed in laboratory experiments^15^,^19^,^20^,^21^,^22^,^32^. A meta-analysis found that the punishment effect was slightly stronger than the reward effect, and the centralization of incentives did not moderate the effect size^32^. Although a self-regarding model that is based on NE analysis or evolutionary game dynamics correctly predicts which incentives are better at promoting cooperation^15^,^18^,^25^,^28^,^35^,^37^,^42^, individuals do not play these rational strategies overall^15^,^21^. In fact, most subjects in PGG with incentives tend to lower (or raise) their contributions if they contributed more (or less) than others in the previous round^2^,^15^,^21^. Furthermore, the subjects exhibited different reactions to peer incentives and institutional incentives. In the peer incentive scenario, both reward and punishment encouraged the recipients to increase contributions^21^. However, in the institutional incentive scenario, only punishment successfully caused the receivers to increase their contributions, and the subjects who were rewarded by the institution often decreased their contributions in the next round^15^. As a result, the contribution levels in institutional reward experiments were not significantly above the standard PGG^15^.

The above discussion suggests that it is important to recognize that actual people are not perfectly rational, and models that are based on self-interest may fail to predict the effectiveness of incentives at promoting cooperation. However, it is unclear how people make decisions when confronted with institutional incentives and why subjects have different attitudes on reward and punishment. In this study, we analyze the observed outcomes in PGG experiments with institutional incentives^15^ and provide an explicit description of human decision making. As an extension of Fischbacher and Gächter’s model^17^, we emphasize that the subjects in the experiments considered not only self-interests but also the behavior of others and whether they were rewarded or punished in the previous round. Our main goal is to answer how the self-regarding preference and the other-regarding preference affect contributions in PGG with institutional incentives and why the reactions to rewards and punishment are different. Interestingly, we find that people on average do not change their behavioral patterns after they are rewarded or punished. In fact, the people who received punishment (or reward) generally contributed less (or more) than their group members, and they increase (or decrease) their contribution in the next round because of the other-regarding preference. Furthermore, the other-regarding preference seems to be independent of the incentive schemes, and institutional incentives promote cooperation by affecting the self-regarding preference.

## Results

### Experimental setups and primary results

The experimental setups and primary experimental results were reported in our previous study^15^, and here, we briefly review them. In the experiments, the subjects interacted anonymously for 50 rounds of the repeated game among the same four players. The control experiment (Control) is a standard four-player repeated PGG. In each round, every subject receives 20 monetary units and decides how much to contribute to the public pool. The total contributions in the pool are then multiplied by 1.6 and split evenly among the four group members. In the nine treatment experiments, each round of PGG is followed by a second stage, which corresponds, respectively to an institutional punishment (IP), an institutional reward (IR), or both institutional reward and punishment (IRP). In a round, exactly one player will be chosen to be rewarded or punished according to his/her contribution (see Methods). For each IR, IP and IRP, there are three different types of incentive intensities, which are called Const, Up and Down, where the amount of punishment/reward is fixed at 20 monetary units in Const, and increases linearly from 16 to 25.6 (or decreases from 25.6 to 16) monetary units per round as a function of the group’s total contribution in Up (or Down).

**Figure 1:**
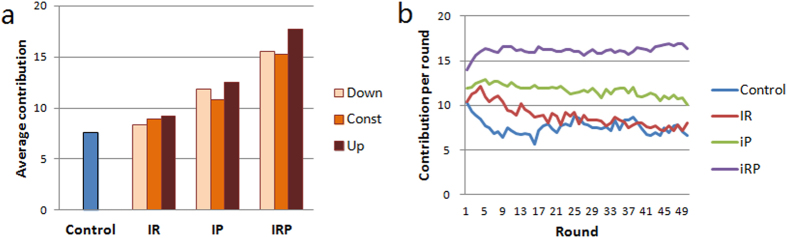
Average contribution in Control and treatment experiments. (**a**) The average contribution for the 50-round sessions in the Control and the nine treatments. The average contributions in IR are 7.55 in Control, 8.32 in Down, 8.95 in Const and 9.18 in Up; in IP, they are 11.80 in Down, 10.79 in Const and 12.55 in Up, and in IRP, they are 15.52 in Down, 15.23 in Const and 17.69 in Up. (**b**) The time evolution of the average contribution per round in Control, IR, IP and IRP. The average contribution significantly increases significantly in IRP, deceases slightly in IP, and decreases significantly in Control and IR.

The primary experimental results are shown in Fig. 1 (the related statistics can be found in Wu *et al*.^15^). For all three types of incentive intensities, IRP is significantly better than either IP or IR in promoting cooperation, and IP has contribution levels significantly above Control, whereas the levels in IR are not significantly above Control (see Fig. 1a). Furthermore, there is a significant increase in contribution levels from the first to the last round in IRP, whereas there is a significant decrease in IR and Control, and a slight decrease in IP (see Fig. 1b). Our previous study showed that although the motivations that are based on (single-round) Nash equilibria correctly predict the evolutionary direction, individuals overall do not play rational strategies^15^. In contrast, in all ten experiments, the proportion of conforming behaviors (i.e., changing the contribution in the next round in the direction of the average group contribution in the current round) is higher than one-half. Furthermore, significantly more individuals increase their contribution after being punished than after being rewarded. However, the correlation between conforming behaviors and reactions to incentives is unknown, and an explicit description of the subjects’ decision making is lacking. Thus, the findings in Wu *et al*.^15^ cannot be applied to evaluate the efficiencies of different incentive schemes on promoting cooperation.

### Modeling human behavior in PGG with institutional incentives

Following Fischbacher and Gächter^17^, we consider that the contribution of a player in PGG with institutional incentives is affected by four factors, which are his/her own behavior, the behavior of others, and whether he/she was rewarded and/or punished in the previous round. Write the contribution of a player in round *t* as


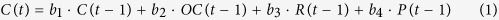


In Eq. (1), *C*(*t* −1) and *OC*(*t* −1) denote the contribution of the player and the average contribution of his/her three other group members, respectively, in round *t* −1. Thus, *b*_1_ and *b*_2_ measure the effects of the self-regarding preference and the other-regarding preference, respectively (see SI Section 2 for a detailed discussion), where *b*_2_ = 0 represents individuals who consider only their self-interests, and *b*_1_ = 0 represents individuals who care only about others’ behavior. In contrast, *R*(*t* −1) and *P*(*t* −1) are the amounts of reward and punishment, respectively, that are received in round *t* −1, and *R*(*t* −1) (or *P*(*t* −1)) equals 0 if the player was not rewarded (or punished). Therefore, *b*_3_ and *b*_4_ descri*b*e the reactions of being rewarded or punished, respectively, where a player tends to increase his/her contribution after being rewarded (or punished) if *b*_3_ > 0 (or *b*_4_ > 0).

Following Eq. (1), the behavioral patterns of the players in the repeated PGG with institutional incentives can be characterized by a 4-dimensional vector (*b*_1_, *b*_2_, *b*_3_, *b*_4_), where free-riders, unconditional contributors and conditional cooperators (who move toward the average contribution of others^10^,^15^,^16^) are denoted by *b*_1_ = 0, *b*_2_ = 0, *b*_1_ = 1, *b*_2_ = 0 and *b*_1_ + *b*_2_ = 1, *b*_2_ > 0, respectively. In particular, the imperfect conditional cooperators who are defined by Fischbacher and Gächter^17^ satisfy *b*_1_ + *b*_2_ < 1 and *b*_2_ > 0. We then calculate the behavioral patterns of the 792 participants based on the regression equation, Eq. (1). The regression results are significant for 79.5% of the players (F-test, P-value < 0.01); furthermore, the results are significant for 91.3% of the players at the 5% significance level. These results imply that the behavior of most individuals in PGG with institutional incentives can be described by our model.

### The effects of reward and punishment

Based on the regression results, we first examine the reactions to reward and punishment. Surprisingly, Table 1 shows that in all nine treatment experiments, the mean values of *b*_3_ and *b*_4_ are very small. Notice that the absolute values are less than 0.05 in all treatments, and the resulting change in the contribution is less than 1 monetary unit. Furthermore, in almost all of the treatments, *b*_3_ and *b*_4_ are not significantly different from zero (the only exception is *b*_3_ in the IRP Const experiment). This finding means that people on average do not react to reward or punishment.

Because *b*_3_ and *b*_4_ are small and their impacts on contributions are not significant, we drop them from Eq. (1) and consider the following simplified regression equation


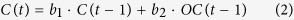


In SI Table S1, we show that excluding *b*_3_ and *b*_4_ from Eq. (1) does not significantly affect the self-regarding parameter *b*_1_ and the other-regarding parameter *b*_2_. In addition, the regression results that are based on Eq. (2) are even better than the results of Eq. (1), i.e., the results are significant for 87.2% of all players (F-test, P-value < 0.01); furthermore, the results are significant for 95.8% of the players at the 5% significance level. This outcome further demonstrates that people on average do not change their behavioral patterns after they are rewarded or punished, and a combination of the self-regarding preference and the other-regarding preference sufficiently explains the dynamics of human behavior in PGG with institutional incentives.

### The formation of human behavior

Based on Eq. (2), we first test whether individuals are sensitive to different types of incentive intensities. For each IR, IP and IRP, we compare *b*_1_ (or *b*_2_) in Up, Const and Down. SI Table S2 shows that in each incentive scheme, there is no significant difference between *b*_1_ (or *b*_2_) in the three incentive intensities, i.e., small changes in the amount of reward or punishment do not affect *b*_1_ and *b*_2_. We therefore combine the data in Up, Const and Down and investigate *b*_1_ and *b*_2_ in the four schemes of Control, IR, IP and IRP.

As shown in Table 2, the mean values of *b*_2_ are between 0.52 and 0.55 in all four schemes, and the differences among them are not significant (see SI Table S3 for a statistical test). In contrast, we observe that *b*_1_ in IRP and IP is larger than in IR and Control, and *b*_1_ in IRP is slightly larger in IP, whereas the difference between *b*_1_ in IR and Control is not significant (see SI Table S3 for a statistical test). We also estimated *b*_1_ and *b*_2_ in the four schemes separately for rounds 1 to 25 and rounds 26 to 50. In IR, IP and IRP, the estimated coefficients are very similar in both halves of the experiments (see SI Table S4). This result means that individual behavioral patterns do not change over rounds in PGG with incentives. However, in Control, *b*_2_ in the first 25 rounds is significantly larger than in the last 25 rounds, whereas *b*_1_ in the first 25 rounds is (insignificantly) smaller than in the last 25 rounds. These findings are consistent with the observation of Fischbacher and Gächters^17^ that belief in others plays a major role in early periods, and self-interest becomes more important later.

These regression results raise two additional questions, namely, why *b*_1_ in IRP and IP is larger than in IR and Control and why *b*_2_ in different incentive schemes is similar. We answer these questions by investigating the correlations between the group average payoff and the group average, *b*_1_, *b*_2_ and *b*_1_ + *b*_2_. The main results are shown in Table 3. In the three incentive schemes of IR, IP and IRP, the correlation between *b*_2_ and the payoff is not significant, whereas we observe a strong positive correlation between *b*_1_ and the payoff in IP and IRP. Thus, a larger *b*_1_ is preferred in IRP and IP because it can lead to a higher payoff. In Control, there is a strong positive correlation between *b*_2_ and the group average payoff, i.e., the level of group average contribution increases in *b*_2_. This finding is consistent with the previous study that conformity promotes the evolution of cooperation in PGG^16^.

**Figure 2:**
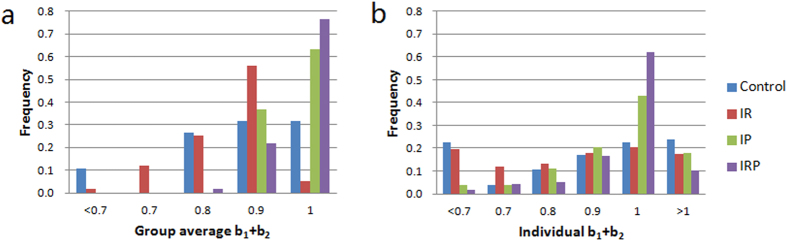
Distribution of *b*_1_ + *b*_2_ in Control, IR, IP and IRP. (**a**) The distribution of the group average, *b*_1_ + *b*_2_. Cooperation can be maintained in groups with *b*_1_ + *b*_2_ ≈ 1. (**b**) The distribution of the individual *b*_1_ + *b*_2_. Most subjects in IRP are conditional cooperators (i.e., *b*_1_ + *b*_2_ ≈ 1), whereas there are many imperfect conditional cooperators (i.e., *b*_1_ + *b*_2_ < 1) in IR, IP and Control.

Now, we explain why the average contributions in IRP and IP are higher than in IR and Control. The analysis in SI Section 2 shows that the group average contribution in a repeated PGG increases in the group average, *b*_1_ + *b*_2_. Furthermore, a high level of contributions can be maintained in a PGG if the group average of *b*_1_ + *b*_2_ ≈ 1 and *b*_2_ are not too small (i.e., the group consists of conditional cooperators^16^). Thus, IRP and IP are better at promoting cooperation than IR and Control because they have a larger *b*_1_ + *b*_2_. Notice that most groups in IRP and IP satisfy *b*_1_ + *b*_2_ ≈ 1, and cooperation increases or does not change significantly in these two incentive schemes (see Fig. 2a). However, *b*_1_ + *b*_2_ < 1 in most groups of IR and Control. As a result, contributions drop significantly in these two schemes.

We also examine *b*_1_ + *b*_2_ at an individual level (see Fig. 2b). Table 2 shows that *b*_1_ + *b*_2_ is significantly smaller than 1 in IR and IP, which means that many subjects in IR and IP are imperfect conditional cooperators. This result explains why cooperation slightly decreases in IP although punishment successfully eliminates the free-riders from the population. Interestingly, individual *b*_1_ + *b*_2_ is insignificantly different from one in Control, which implies that many people in Control are conditional cooperators. As shown in Fig. 2b, individual behavioral patterns in Control have a large degree of heterogeneity, where free-riders (i.e., *b*_1_ + *b*_2_ ≪ 1) and conditional cooperators (i.e., *b*_1_ + *b*_2_ ≈ 1,*b*_2_ > 0) are the two largest types. Thus, cooperation can be maintained in groups that consist of conditional cooperators, and contributions drop if there are free-riders or imperfect conditional cooperators in the group (as shown in Fig. 2a, approximately 30% of the groups in Control comprise conditional cooperators).

## Discussion

Recent empirical research has shown that conditional cooperation or conforming behaviors are common in PGG experiments^8^,^9^,^10^,^11^,^12^,^13^,^14^,^15^, and people may have different attitudes concerning reward and punishment^15^,^19^,^20^,^21^,^22^,^32^,^45^. The goal of our study is to explore how the self-regarding preference and the other-regarding preference affect contributions in repeated PGG with institutional incentives and why some incentive schemes promote cooperation better than others. To achieve this goal, we consider that individual contributions are affected by four factors, which are self-interest, the behavior of others, and the reactions to reward and punishment. The regression results show that people on average do not react to reward or punishment and that two factors, namely, self-interest and the behavior of others, sufficiently explain the dynamics of human behavior. Furthermore, institutional incentives promote contributions by affecting the self-regarding preference, *b*_1_, and the other-regarding preference, *b*_2_, seems to be independent of incentive schemes.

Our conclusion questions the applicability of many theoretical models of PGG with institutional incentives^15^,^31^,^34^,^35^,^36^,^37^,^38^,^39^,^40^,^41^,^42^,^43^,^44^. The evolutionary/learning dynamics that are considered in these models are based on the assumptions of perfect rationality, such as Nash equilibrium strategies (or best response strategies)^15^,^35^,^39^, or preferential imitation of better performing players, such as replicator dynamics^35^,^37^,^40^,^41^,^42^,^43^, pairwise comparison updating^34^,^36^,^38^,^44^, and exploration dynamics^31^,^39^. However, our experiments did not provide significant evidence that the subjects choose payoff maximizing strategies or imitate their group members with the best payoff. Instead, the contributions of the players mainly depend on their own previous action and the actions of their group members. Accordingly, we suggest that subsequent theoretical research on PGG with institutional incentives should consider our findings that players seem to not care about the payoffs of their group members in updating their actions, but they care about their group members’ contributions.

Our analysis also reveals that individuals display different behavioral patterns when confronted with institutional incentives and peer incentives. The experiments on peer incentives have shown that people behave more cooperatively after being rewarded or punished^21^,^22^,^39^. These observations indicate a problem with peer incentives: the maintenance of cooperation by peer incentives relies on whether defectors are punished and cooperators are rewarded in time, and the cooperation level will decline if the reward or punishment level declines. It has been shown that the average number of peer punishers (or rewarders) decreases with the number of defectors (or cooperators)^21^,^39^. This result implies that peer incentives are inherently fragile because reward levels are difficult to maintain in a cooperative population and because punishment levels are difficult to maintain in a selfish population. In contrast, in PGG with institutional incentives, because individuals do not change their behavioral patterns regardless of whether they received incentives, the mere potential to punish defectors and to reward cooperators can lead to considerable increases in the level of cooperation. In our experiments, only one player will be rewarded or punished in each round. Therefore, institutional incentives are more powerful than peer incentives in promoting cooperation because the incentive institutions work although not all defectors are punished.

Finally, it is well known that human populations are in general highly structured, where different individuals interact with different subsets of the entire population. Theoretical studies that are based on evolutionary game methods have indicated that structures play a major role in the evolution of cooperation in social dilemma games, such as prisoner’s dilemma (PD) and PGG^46^,^47^,^48^,^49^,^50^,^51^,^52^,^53^,^54^,^55^. However, cooperative outcomes have been rarely observed in experiments on many static networks^56^,^57^,^58^,^59^,^60^. A possible explanation for this inconsistency is that most theoretical studies have considered payoff-based updating rules, whereas the people in the experiments did not adopt these rules^61^,^62^,^63^. In particular, a behavioral rule called “moody conditional cooperation” was observed in several spatial PD game experiments, where moody conditional cooperators make decisions based on their own previous action and the actions of their neighbors (specifically, they cooperate more when they themselves cooperated in the previous round and more of their neighbors cooperated)^61^,^63^. There is a direct connection between moody conditional cooperation and our model^16^, and our method can also be applied to describe the dynamics of human behavior on networked PD games. By introducing network parameters into the model (e.g., the number of neighbors), we can expect to quantitatively evaluate the effect of network structures on human decision making.

## Methods

A total of 792 university students participated in our PGG experiments at the School of Mathematical Sciences Computer Lab at Beijing Normal University. All participants provided written informed consent after the nature and possible consequences of the studies were explained. All experimental methods were conducted according to the approved guidelines. All experimental protocols were approved by the Ethics Review Committee of the Institute of Zoology.

The subjects interacted anonymously through computer screens for 50 rounds of the repeated game among the same four players. The control experiment (Control, 76 subjects, 19 groups) is a standard four-player repeated PGG. In the treatment experiments, each round of PGG is followed by an incentive stage, which corresponds to an institutional punishment (IP), an institutional reward (IR), or both institutional reward and punishment (IRP). Furthermore, for each IR, IP and IRP, there are three different types of incentive intensities that are called Const, Up and Down. In IP, there are 80 subjects (20 groups) in Const, 76 subjects (19 groups) in Up and 84 subjects (21 groups) in Down. In IR, there are 80 subjects (20 groups) in Const, 72 subjects (18 groups) in Up and 84 subjects (21 groups) in Down. In IRP, there are 80 subjects (20 groups) in Const, 80 subjects (20 groups) in Up and 80 subjects (20 groups) in Down.

In Control, an individual’s single round expected payoff is 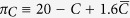 when he/she contributes *C* and the average contribution of the group is 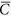. In the treatments, exactly one player will be chosen to be rewarded or punished refers according to his/her contribution in the incentive stage. An individual’s expected payoffs are *π*_*IR*_ = *π*_*C*_ + *P*_*IR*_*A* in IR, *π*_*IP*_ = *π*_*C*_ −*P*_*IP*_*A* in IP, and *π*_*IRP*_ = *π*_*C*_ + (*P*_*IR*_−*P*_*IP*_)*A* in IRP, where *A* denotes the amount of incentives (which is *A* = 20 in Const, 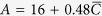 in Up and 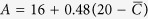 in Down), and *P*_*IR*_ (or *P*_*IP*_) is the probability that the individual is rewarded in IR (or punished in IP). Specifically, 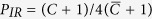, and 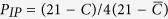. Thus, the probability that an individual is rewarded (or punished) increases (or decreases) as the amount that he/she contributed increases. More methodological details and sample instructions can be found in Wu *et al*.^15^.

## Additional Information

**How to cite this article**: Dong, Y. *et al*. The dynamics of human behavior in the public goods game with institutional incentives. *Sci. Rep*. **6**, 28809; doi: 10.1038/srep28809 (2016).

## Supplementary Material

Supplementary Information

## Figures and Tables

**Table 1:** Reactions to reward and punishment.

		Up	Const	Down
IR	*b*_3_	0.0443	0.0492	0.0430
IP	*b*_4_	−0.0062	0.0352	0.0274
IRP	*b*_3_	0.0115	0.0415	0.0392*
	*b*_4_	0.0119	0.0142	−0.0027

The mean values of *b*_3_ and *b*_4_ in the nine treatment experiments. The symbol “^*^” denotes that the mean value of *b*_3_ is significantly different from zero (Mann-Whitney U-test, P-value < 0.01). The data are analyzed at the group level to avoid the interdependence of outcomes for members of a given group.

**Table 2:** The mean values of *b*_1_ and *b*_2_ in Control, IR, IP and IRP.

	Control	IR	IP	IRP
*b*_1_	0.3332	0.3235	0.4026	0.4373
*b*_2_	0.5421	0.5264	0.5518	0.5314
*b*_1_ + *b*_2_	0.8753	0.8499*	0.9544*	0.9687

The symbol “*” denotes that the individual *b*_1_ + *b*_2_ is significantly smaller than one (Mann-Whitney U-test, P-value < 0.01). Most people in IR and IP are imperfect conditional cooperators (i.e., *b*_1_ + *b*_2_ < 1), whereas most people in IRP are conditional cooperators (i.e., *b*_1_ + *b*_2_ ≈ 1).

**Table 3:** Correlation coefficients between the group average payoff and the group average, *b*_1_, *b*_2_ and *b*_1_ + *b*_2_ in Control, IR, IP and IRP.

	Control	IR	IP	IRP
*b*_1_	−0.0006	0.1678	0.3795*	0.4036*
*b*_2_	0.7173*	−0.0503	−0.2663	−0.2853

The symbol “*” denotes that the correlation is strong, i.e., P-value < 0.01.
