# Supplementary material for: The dynamics of human behavior in the public goods game with institutional incentives
Source: Sci Rep. 2016 Jun 24;6:28809. doi: 10.1038/srep28809 (PMC4919618; doi:10.1038/srep28809)
Supplement: Supplementary Information [file srep28809-s1.doc]

**Supplementary information for “The dynamics of human behavior in the public goods game with institutional incentives”**

**Yali Dong^1^, Boyu Zhang^2^, Yi Tao^3^**

^1^School of Statistics, Beijing Normal University, Beijing, China

^2^Laboratory of Mathematics and Complex Systems, Ministry of Education, School of Mathematical Sciences, Beijing Normal University, Beijing, China

^3^Key Lab of Animal Ecology, Institute of Zoology, Chinese Academy of Sciences, Beijing, China

^*^Authors for correspondence: [zhangby@bnu.edu.cn](mailto:zhangby@bnu.edu.cn)

1. **Statistical analysis**

In Tables S1-S4, the data is analyzed at the group level to avoid interdependence of outcomes for members of a given group. In Control, there are 19 groups; in IP, there are 20 groups in Const, 19 groups in Up and 21 groups in Down; in IR there are 20 groups in Const, 18 groups in Up and 21 groups in Down; in IRP there are 20 groups in Const, 20 groups in Up and 20 groups in Down.

**Table S1 Comparisons between regression coefficients (i.e., $b_{1}$ and $b_{2}$) of Eq.(1) and Eq.(2) in each of the nine treatment experiments. Notice that P-values of Mann-Whitney U-test is large than 0.01 for all incentive schemes and all incentive intensities, excluding $b_{3}$ and $b_{4}$ from Eq.(1) does not significantly affect $b_{1}$ and $b_{2}$.**

|  |  | Up | Const | Down |
| --- | --- | --- | --- | --- |
| IR | $b_{1}$ | 0.4383 | 0.4094 | 0.3024 |
|  | $b_{2}$ | 0.9118 | 0.8392 | 0.7436 |
| IP | $b_{1}$ | 0.9767 | 0.9031 | 0.5800 |
|  | $b_{2}$ | 1.0000 | 0.4735 | 0.3651 |
| IRP | $b_{1}$ | 0.5979 | 0.5609 | 0.7972 |
|  | $b_{2}$ | 0.8181 | 0.9892 | 1.0000 |

**Table S2** Comparisons between regression coefficients (i.e., $b_{1}$ and $b_{2}$) of the three different incentive intensities in each of the three incentive schemes. Notice that P-values of Mann-Whitney U-test is large than 0.01 for all incentive schemes, individuals are not sensitive to different types of incentive intensities.

|  |  | Up vs Down | Up vs Const | Down vs Const | |
| --- | --- | --- | --- | --- | --- |
| IR | $b_{1}$ | 0.7674 | 0.3420 | | 0.3966 |
|  | $b_{2}$ | 0.8107 | 0.8264 | | 0.9066 |
| IP | $b_{1}$ | 0.8496 | 0.9664 | | 0.8245 |
|  | $b_{2}$ | 0.5157 | 0.9664 | | 0.6110 |
| IRP | $b_{1}$ | 0.4407 | 0.6554 | | 0.6554 |
|  | $b_{2}$ | 0.7972 | 0.5250 | | 0.6168 |

**Table S3** Comparisons between regression coefficients (i.e., $b_{1}$ and $b_{2}$) of the four schemes, Control, IR, IP and IRP. The symbol “*” denotes that the difference is significant (Mann-Whitney U-test, P-value<0.01). The differences between $b_{2}$ in all the four schemes are not significant. In contrast, $b_{1}$ in IRP and IP are significantly larger than IR, and insignificantly larger than Control. Furthermore, $b_{1}$ in IRP is slightly above IP, while the difference between $b_{1}$ in IR and Control is not significant.

|  | IP vs IR | IP vs IRP | IR vs IRP | IP vs C | IR vs C | IRP vs C |
| --- | --- | --- | --- | --- | --- | --- |
| $b_{1}$ | 0.0030* | 0.1135 | <0.001* | 0.0679 | 0.9841 | 0.0075* |
| $b_{2}$ | 0.1974 | 0.3597 | 0.7884 | 0.6787 | 0.8714 | 0.8833 |

**Table S4** Estimations of $b_{1}$ and $b_{2}$ in the four schemes, Control, IR, IP and IRP, for rounds 1 to 25 and rounds 26 to 50. The symbol “*” denotes that the difference is significant (Mann-Whitney U-test, P-value<0.01). In IR, IP and IRP, mean values of $b_{1}$ (or $b_{2}$) are very similar in both halves of the experiment. In Control, $b_{2}$ in the first 25 rounds is significantly larger than the last 25 rounds, while $b_{1}$ in the first 25 rounds is (insignificantly) smaller than the last 25 rounds.

|  |  | Rounds 1-25 | Rounds 26-50 | P-value | |
| --- | --- | --- | --- | --- | --- |
| Control | $b_{1}$ | 0.2627 | 0.3640 | | 0.2672 |
|  | $b_{2}$ | 0.6149 | 0.4514 | | 0.0079* |
| IR | $b_{1}$ | 0.2860 | 0.2669 | | 0.5538 |
|  | $b_{2}$ | 0.5800 | 0.5425 | | 0.4909 |
| IP | $b_{1}$ | 0.3722 | 0.3767 | | 0.8317 |
|  | $b_{2}$ | 0.5892 | 0.5537 | | 0.4770 |
| IRP | $b_{1}$ | 0.4348 | 0.5408 | | 0.8276 |
|  | $b_{2}$ | 0.4376 | 0.5083 | | 0.5549 |

1. **Theoretical analysis**

Consider that players are boundedly rational and update their behavior in a 4-player repeated PGG according to the conditional cooperation rule Eq.(2). Let us now investigate under what conditions group average contribution does not decrease over rounds. Denote the contribution of player $i$ ($i=1,2,3,4$) in round $t$ by $C_{i}(t)$. In a homogeneous population where all four players in the PGG have the same $b_{1}$ and $b_{2}$ , we have

$\begin{matrix} C_{i}\left(t\right)=b_{1}\cdot C_{i}\left(t-1\right)+b_{2}\cdot\frac{\sum_{j\neq i} C_{j}\left(t-1\right)}{3} \\ =(b_{1}+b_{2})C_{i}\left(t-1\right)+\frac{4b_{2}}{3}\left(\overline{C}(t-1)-C_{i}(t-1)\right). \end{matrix}$ (S1)

Thus, the group average contribution in round $t$ (denoted by $\overline{C}(t)$) can be written as

$\overline{C}(t)=\sum_{i=1}^{4} C_{i}\left(t\right)/4=(b_{1}+b_{2})\overline{C}(t-1)$. (S1)

On the other hand, in a heterogeneous population where players in the PGG have different behavioral patterns (denote the behavioral pattern of player $i$ by $(b_{i1},b_{i2})$ with $i=1,2,3,4$), we have

$\begin{matrix} C_{i}\left(t\right)=b_{i1}\cdot C_{i}\left(t-1\right)+b_{i2}\cdot\frac{\sum_{j\neq i} C_{j}\left(t-1\right)}{3} \\ =(b_{i1}+b_{i2})C_{i}\left(t-1\right)+\frac{4b_{i2}}{3}\left(\overline{C}(t-1)-C_{i}(t-1)\right), \end{matrix}$ (S3)

and the group average contribution in round $t$ is written as

$\overline{C}(t)=\sum_{i=1}^{4} C_{i}\left(t\right)/4=\frac{\sum_{i=1}^{4} (b_{i1}+b_{i2})C_{i}\left(t-1\right)}{4}+\frac{\sum_{i=1}^{4} b_{i2}\overline{C}(t-1)-\sum_{i=1}^{4} b_{i2}C_{i}\left(t-1\right)}{3}$ (S4)

Furthermore, if four players contribute the same (i.e., $C_{i}(t-1)=\overline{C}(t-1)$ for all $i=1,2,3,4$) in round $t-1$, Eq.(S4) can be simplified as $\overline{C}(t)=(\overline{b_{1}}+\overline{b_{2}})\overline{C}(t-1)$, where $\overline{b_{1}}$ and $\overline{b_{2}}$ are the group average $b_{1}$ and $b_{2}$, respectively . The above analysis implies that $\overline{b_{1}}+\overline{b_{2}}$ plays a crucial role in maintaining cooperation, group average contribution in a repeated PGG is increasing in group average $b_{1}+b_{2}$. In particular, in a homogeneous population or in a heterogeneous population where players have different behavioral patterns but the same contribution level, group average contribution does not decrease over rounds if and only if $\overline{b_{1}}+\overline{b_{2}}\geq1$.
